# Cyclooxygenase Inhibition Safety and Efficacy in Inflammation-Based Psychiatric Disorders

**DOI:** 10.3390/molecules25225388

**Published:** 2020-11-18

**Authors:** Maria Grazia Perrone, Antonella Centonze, Morena Miciaccia, Savina Ferorelli, Antonio Scilimati

**Affiliations:** Department of Pharmacy-Pharmaceutical Sciences, University of Bari, Via E. Orabona 4, 70125 Bari, Italy; mariagrazia.perrone@uniba.it (M.G.P.); antonella.centonze1@uniba.it (A.C.); morena.miciaccia@uniba.it (M.M.); savina.ferorelli@uniba.it (S.F.)

**Keywords:** major depression, bipolar disorder, schizophrenia, autism spectrum disorder, cyclooxygenase (COX) inhibitors

## Abstract

According to the World Health Organization, the major psychiatric and neurodevelopmental disorders include major depression, bipolar disorder, schizophrenia, and autism spectrum disorder. The potential role of inflammation in the onset and progression of these disorders is increasingly being studied. The use of non-steroidal anti-inflammatory drugs (NSAIDs), well-known cyclooxygenase (COX) inhibitors, combined with first-choice specific drugs have been long investigated. The adjunctive administration of COX inhibitors to classic clinical treatments seems to improve the prognosis of people who suffer from psychiatric disorders. In this review, a broad overview of the use of COX inhibitors in the treatment of inflammation-based psychiatric disorders is provided. For this purpose, a critical analysis of the use of COX inhibitors in the last ten years of clinical trials of the major psychiatric disorders was carried out.

## 1. Introduction

**Figure 1:**
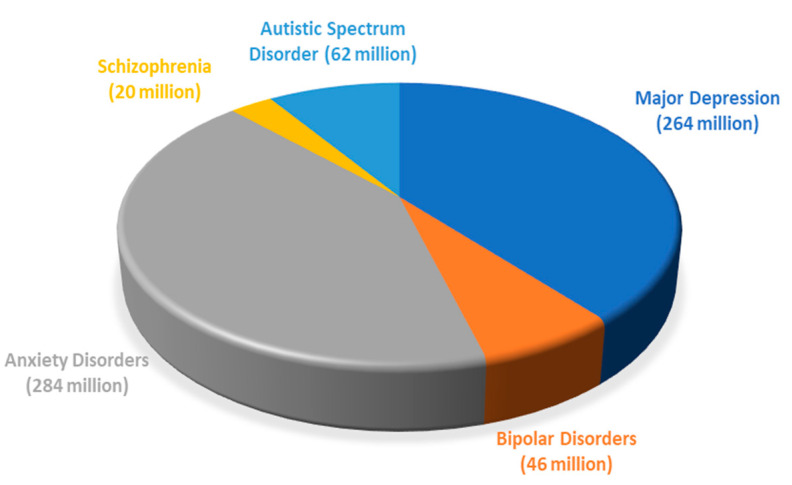
The five most common psychiatric disorders worldwide.

According to the American Psychiatric Association, psychiatric disorders, also called mental disorders or mental illnesses, are defined as health conditions involving changes in emotion, thinking, or behavior (or a combination of these manifestations) [1]. According to the World Health Organization (WHO), the most common mental illnesses include major depression, bipolar disorder, schizophrenia, and other psychoses, dementia, and developmental disability [2]. All these disorders affect more than 600 million people worldwide, distributed as 264 million affected by major depression, 46 million with bipolar disorders, 284 million with anxiety disorders, 20 million people with schizophrenia, and 62 million show autistic spectrum disorder (Figure 1) [2]. Psychiatric disorders usually occur for the first time in childhood and/or adolescence, however, the treatment but not cure of these illnesses is often done several years later [3].

Although monoaminergic dysregulation is the prevalent hypothesis of the pathogenesis of neuropsychiatric disorders, the existence of refractory patients to the monoamine oxidase inhibitors treatment sets limits on this assumption. Thus, the existence of other putative mechanisms underlying these diseases has been inquired about over the years. Experimental evidence has revealed that inflammation, a protective response of the immune system to pathogens or tissue damage caused by chemical, physical or biological agents, might also play a key role in several mental disorders [4,5]. Furthermore, the inflammatory response in the brain, called “neuroinflammation”, can be triggered not only by pathogen infections, traumatic brain injury, toxic metabolites, and autoimmunity, but also by psychological stress [6,7].

## 2. Role of COX Inhibitors in Mental Disorders

Patients affected by neuropsychiatric disorders show all features of inflammation, including increased circulating levels of inflammatory inducers such as pathogen-associated molecular patterns (PAMPs) and damage-associated molecular patterns (DAMPs) [8]. PAMPs are molecules or parts of molecules (e.g., lipopolysaccharide, double-stranded viral RNA, unmethylated CpG sequences) released by some pathogens not expressed by the host organism, identified as non-self by the cells of the innate immune system. DAMPs are endogenous molecules that increase cellular and oxidative stress, exposure to stressful factors, and tissue damage. Examples of DAMPs are extracellular ATP, circulating uric acid, heat shock proteins, high mobility group box 1, and oxidized molecules. All of the above molecules are induced by psychosocial stress and are capable of activating both central and peripheral inflammatory responses [8,9,10,11,12]. Innate immune cells (microglia, astrocytes, and oligodendroglia) through to macrophages, monocytes, dendritic cells, and mast cells (but also non-immune cells such as epithelial cells and fibroblasts), express pattern recognition receptors (PRR). These include Toll-like receptors (TLRs), cytoplasmic NOD-like receptors, intracellular retinoic acid-inducible gene-I-like receptors (RLR), transmembrane C-type lectin receptors. PAMPs and DAMPs bind PRR, determining concomitant conformational changes that prompt a cascade of downstream signaling resulting in transcriptional changes, as well as post-translational modifications [13].

**Figure 2:**
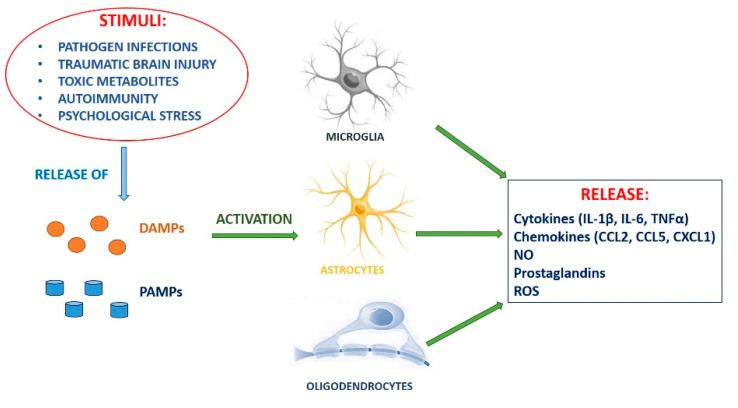
Neuroinflammation: Inflammatory response triggered by pathogen infections, traumatic brain injury, toxic metabolites, autoimmunity, and psychological stress.

Specifically, neuroinflammation by innate immune cells is characterized by the presence in the inflamed cells/tissues of pro-inflammatory cytokines (interleukin (IL)-1β, IL-6, and tumor necrosis factor (TNF)α), chemokines (CCL2, CCL5, CXCL1), secondary inflammatory mediators like nitric oxide (NO) and prostaglandins (PGs), and reactive oxygen species (ROS) (Figure 2) [6,14,15,16,17,18]. The effects of the chronic neuroinflammatory condition could lead to the development of psychiatric diseases. Indeed, major depression, schizophrenia [19,20,21,22,23,24], and bipolar disorder [25,26,27] are associated with a dysregulated immune response, as proven by the abnormal profiles of pro- and anti-inflammatory cytokines observed in affected patients [28].

The central nervous system (CNS) was generally believed to be an immunologically privileged organ since peripheral immune components were not allowed to infiltrate into the CNS. However, nowadays, increasing evidence demonstrates that the immune responses of the CNS are in close communication with those occurring in the periphery [29]. In fact, defective or inappropriate communication between the immune and nervous system is emerging as a common hallmark in several etiologically different CNS diseases, including neurodevelopmental, neurodegenerative, and neuroimmunological disorders. Several mediators and mechanisms have been considered responsible for interacting with the CNS, possibly because each plays a role at some level, and their importance depends on the experimental system being examined. The crosstalk between the peripheral and CNS’ immune components (such as, activated CNS microglia and astrocytes, pro-inflammatory periphery monocytes/macrophages and T lymphocytes, and infiltrated monocytes/macrophages and T lymphocytes, as well as the immunoreactive molecules they release) are closely related to CNS-homeostasis, disease onset and progression. A unique feature of CNS-immune system physiology, however, is that the entire region of the brain parenchyma is excluded from the peripheral immune system and that baseline parenchymal immunity is instead mediated by microglia, the tissue-resident macrophages of the brain [30]. Microglia are able to interact with a variety of CNS immune components as well as peripheral immune components that have infiltrated into the CNS, making themselves the focus of multiple crosstalk events [31].

**Figure 3:**
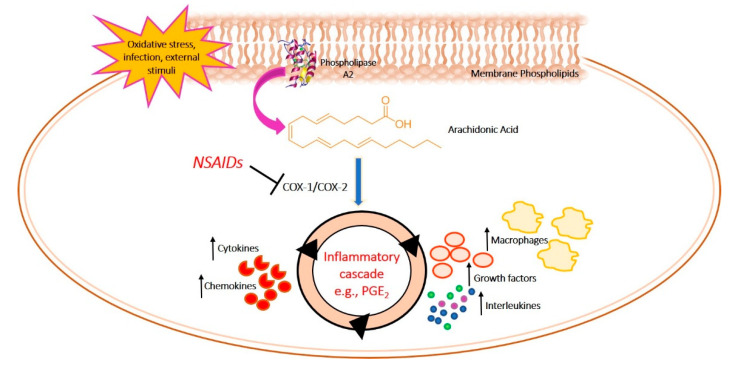
Inflammatory cascade mediated by the COX.

Due to the involvement of chronic inflammation in the onset and progression of psychiatric disorders, the use of anti-inflammatory drugs (such as non-steroidal anti-inflammatory drugs (NSAIDs) like cyclooxygenase (COX) inhibitors) in the treatment of these diseases has been long investigated. COX, also called Prostaglandin H synthase (PGHS) is a key enzyme in the inflammatory cascade. It catalyzes the conversion of arachidonic acid (AA) in prostanoids, bioactive lipids mediating numerous physiological and pathological processes in the body [32]. Prostanoids include thromboxane A_2_ (TXA_2_), prostaglandins (PGD_2_, PGE_2_, PGF_2α_), and prostacyclin (PGI_2_) (Figure 3) [33].

**Figure 4:**
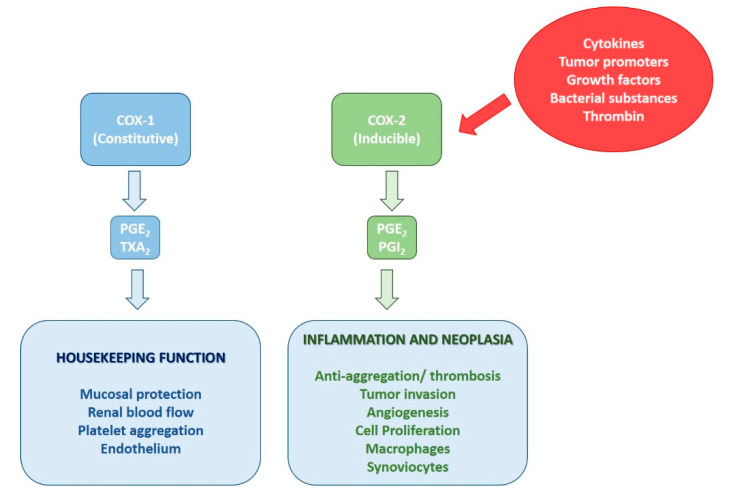
Some functional differences between COX-1 and COX-2 isoforms.

Two COX isoforms are known, COX-1 and COX-2, encoded by different genes. The two isoforms show 60% homology in their amino acid sequence. COX-1 is the isoform constitutively expressed in most tissues and responsible for maintaining the normal physiological functions, such as gastric protection, modulation of platelet function, and renal homeostasis [34]. COX-2, differently from COX-1, is the inducible isoform upon pro-inflammatory stimuli. Its expression can be rapidly induced by cytokines, tumor promoters, growth factors, bacterial substances, and thrombin [33] (Figure 4). It has been found that both isoforms are constitutively expressed in the brain. COX-1 is expressed by microglia and perivascular cells [14,15,16], while COX-2 is identified in post-synaptic dendrites and excitatory terminals, in particular in the cortex, hippocampus, and amygdala [35,36,37]. This underlies the crucial neurological functions in which COX-2 is involved in the CNS, such as synaptic activity, long-term potentiation, long-term depression, and memory consolidation [38,39]. As with microglia, peripheral monocytes/macrophages possess neuroprotective and neurotoxic properties and improving their anti-inflammatory response, inhibiting their pro-inflammatory response using, for example, NSAIDs may be an important future therapeutic strategy to reduce neuroinflammation and therefore the onset of psychiatric disorders [40,41].

Over the years, various studies were carried out to clarify both the involvement of COXs and the effectiveness of their inhibitors in pathologies with a marked inflammatory component [42].

**Figure 5:**
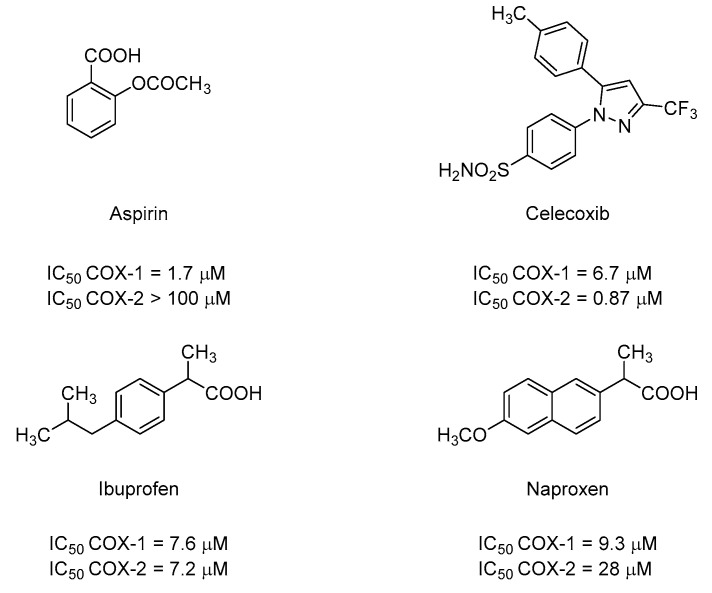
Chemical structure of NSAIDs used in clinical practice.

The purpose of this review is to summarize in detail the outcome of the treatments of psychiatric disorders with COX inhibitors, evaluating the relationship between such disorders and the inflammatory process. NSAIDs (Figure 5) used in the examined studies are:

- Aspirin, which at low-dose (≤100 mg daily) irreversibly inhibits platelet COX-1 with a COX-1 IC_50_ = 1.7 μM and >100 μM for COX-2 measured in a whole blood assay (HWBA) [43].
- Celecoxib, a selective COX-2 inhibitor, inhibits COX-1 with an IC_50_ = 6.7 μM and COX-2 with IC_50_ = 0.87 µM (HWBA) [43,44].
- Ibuprofen, which is a non-preferential COX inhibitor with an IC_50_ of 7.6 μM and 7.2 µM (HWBA) for COX-1 and COX-2, respectively [43].
- Naproxen a preferential COX-1 inhibitor, inhibits COX-1 with an IC_50_ = 9.3 μM and COX-2 with IC_50_ = 28 µM (HWBA) [43].

In general, NSAIDs cross the BBB efficiently, though the effective dose reaching the brain may vary depending on the neuropathological conditions, affecting BBB integrity [45,46].

### 2.1. Major Depressive Disorder (MDD)

Major depressive disorder (MDD), also commonly known as depression, is an affective mood disorder. It is the most representative psychiatric disease [47,48]. Besides, it is a serious illness affecting approximately 17% of the USA population, resulting in highly unfavorable social and economic outcomes [49]. Typical symptoms include sadness, reduced interests and activity, mnestic deficits, sleep, and weight changes [48]. Despite the variety of antidepressant therapies, only one-third of depressed patients show significant improvement in response to first-line treatment [50,51].

The relationship between levels of pro-inflammatory cytokines and depression was first described in 1991 by Smith et al. in the “Macrophage theory of depression” [52]. This theory relied on the observations that cytokines produced by macrophages, when administered to healthy volunteers induced typical symptoms of depression, and showed effects in the brain, including the activation of the hypothalamic-pituitary-adrenal (HPA) axis [53,54]. Afterward, further studies supported the existence of an association between depression and inflammatory processes, a connection that seems to be bidirectional since patients with depression show elevated levels of peripheral pro-inflammatory markers, independent from comorbid somatic illnesses [55,56,57,58,59]. Indeed, depressed individuals show increased blood concentrations of pro-inflammatory cytokines [60], including interleukin-1β (IL-1β) [61], interleukin-6 (IL-6) [55,56,62,63,64], interleukin-18 (IL-18), TNF-α [55,64,65], interferon-gamma (INF-γ), and other acute-phase proteins [37,66], such as C-reactive protein (CRP) [55,56,63,67], haptoglobin [68] and neopterin [56,69].

Depression probably represents a maladaptive version of cytokine-induced sickness, which might occur in the presence of an exacerbation in intensity and/or duration of the innate immune response, or in the case of increased vulnerability to depression [59]. Moreover, depression occurs at a substantially higher rate in patients with inflammatory disorders such as multiple sclerosis, psoriasis, rheumatoid arthritis, inflammatory bowel disease [70,71], myocardial infarction [72].

Three mechanisms on how cytokines may lead to depression or depressive symptoms were hypothesized [47]: (1) the stimulation of the indoleamine-2-3-dioxygenase (IDO) [73], (2) the modulation of the serotoninergic neurotransmission (not IDO-mediated), and (3) the activation of the HPA axis.

The indoleamine-2-3-dioxygenase is an enzyme involved in the metabolism of tryptophan [73], an essential amino acid actively transported into the brain for serotonin biosynthesis [59].

**Figure 6:**
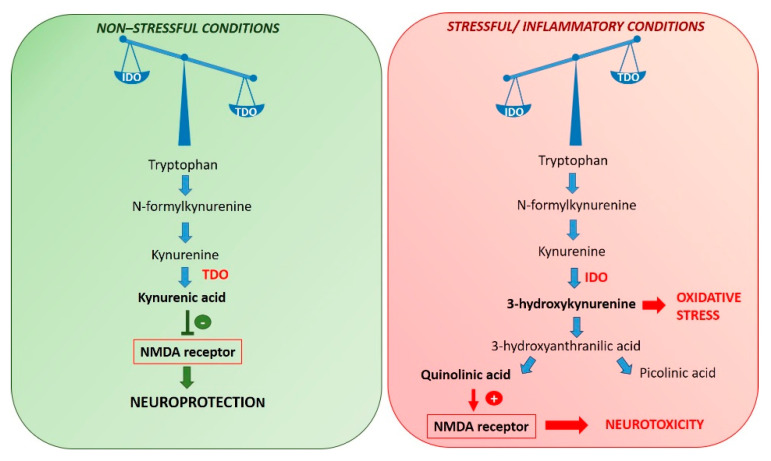
Tryptophan metabolism in both non-stressful and stressful conditions. NMDA (*N*-methyl-d-aspartate).

Tryptophan levels are linked to the pathogenesis of depression because acute tryptophan depletion decreases mood both in vulnerable people with a familial history of MDDs and in drug-free people in remission after an episode of major depression [59,74]. Under non-stressful conditions, tryptophan is metabolized by hepatic tryptophan 2,3-dioxygenase (TDO) to kynurenine, while in stressful and inflammatory conditions, tryptophan is metabolized by IDO [6,75], which is largely distributed in accessory immune cells (such as macrophages and dendritic cells), lung, kidney [76,77], microglia, astrocytes, and neurons [78,79,80]. Both enzymes degrade tryptophan in the kynurenine pathway. However, while TDO is activated by cortisol, IDO can be directly activated by a wide number of pro-inflammatory cytokines, including interferon (IFN)-γ and TNF-α. Hence, the pro-inflammatory cytokines activating IDO reduce the bioavailability of tryptophan, favoring the development of depression. In contrast, anti-inflammatory cytokines reduce the activity of IDO [6] (Figure 6).

Activation of IDO by pro-inflammatory cytokines can induce behavior like depression also through the generation of neuroactive mediators in the kynurenine pathway [59]. Under non-inflammatory conditions, the final product of kynurenine metabolism is kynurenic acid, a neuroprotective metabolite that acts as an antagonist of the NMDA glutamate receptor [81]. On the contrary, as a consequence of the activation of the microglia from stress or inflammation, the neurodegenerative pathway predominates [82], with a consequent increase of levels of quinolinic acid and 3-hydroxy-kynurenine, two excitotoxic metabolites acting as agonists of the NMDA receptor [47,83,84] and inducing neuronal damage via oxidative stress [47,85]. Probably, the net result is an alteration in glutamatergic neurotransmission, that could trigger the depression [6,86].

**Figure 7:**
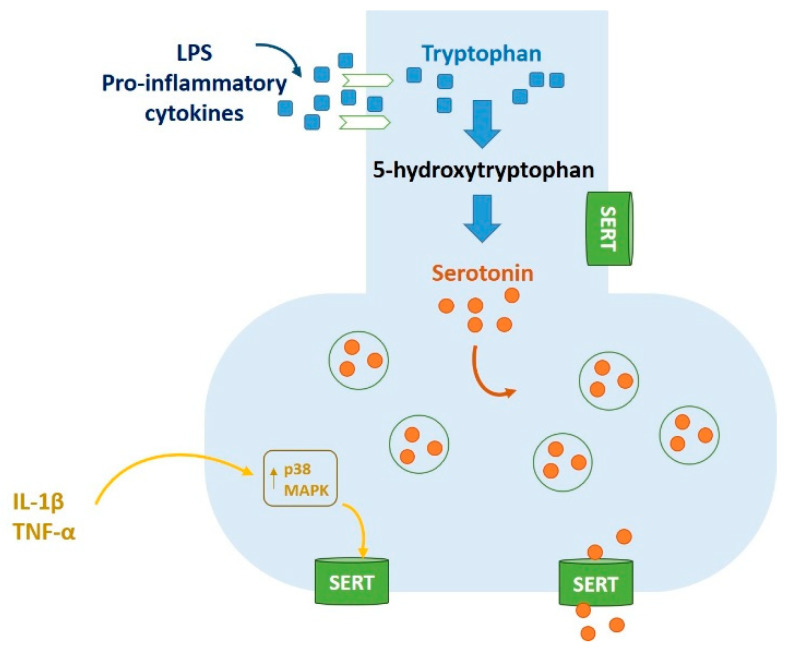
Modulation of the serotoninergic neurotransmission (not IDO-mediated).

Probably, the over-activation of IDO is not the only mechanism involved in the development of inflammation-associated MDD. It was observed that cytokines might modulate serotonergic neurotransmission through different mechanisms. There is evidence that lipopolysaccharides (LPS) and pro-inflammatory cytokines increase tryptophan uptake in the brain and serotonin turnover [87]. Furthermore, Zhu et al. hypothesized that IL-1β and TNF-α might also activate neuronal serotonin transporters (SERT) [88], probably with the p38 MAPK pathway [89] (Figure 7). In addition, IFN-α decreases the expression of serotonin receptor 1A in a variety of non-neuronal cell lines [90].

**Figure 8:**
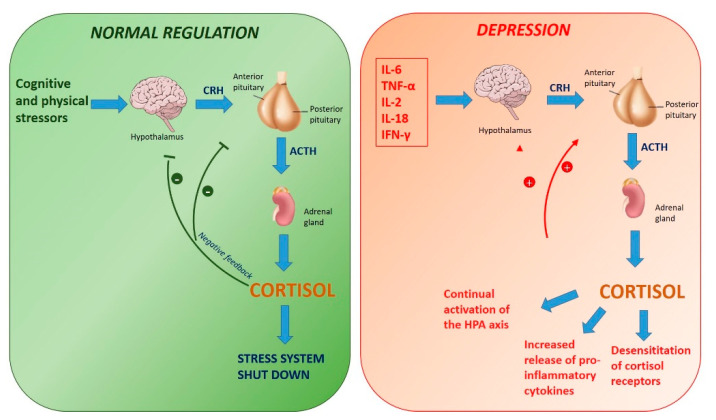
Activation of the hypothalamic-pituitary-adrenal (HPA) axis.

The last hypothesized mechanism is represented by the over-activation of the HPA axis [59,91]. Indeed, it was observed that this axis can be acutely and potently activated by pro-inflammatory cytokines. In particular, pro-inflammatory cytokines induce gene expression and synthesis of corticotrophin-releasing hormone (CRH) which in turn stimulates the release of adrenocorticotropic hormone (ACTH) and causes glucocorticoid secretion [73,92]. An over-activated HPA axis may cause a further increase in pro-inflammatory cytokines, through a positive feedback loop [73]. The mentioned increased inflammatory response in the brain determines a decreased inhibitory feedback on CRH by glucocorticoids, thereby intensifying the stress-response system [93] (Figure 8). Schuld et al. reverted this hypothesis, according to which the long-term over-activation of the HPA axis in patients with depression suppresses the production of inflammatory cytokines [94]. Further studies are necessary to spread light on this mechanism.

Since the inflammatory state is associated with the development and progression of depression, the use of NSAIDs in combination with antidepressants seems to have clinical efficacy in depression due to COX-2 inhibition. NSAIDs exert their antidepressant effects not only through inhibition of COX-2, but also through other mechanisms, including reduction of oxidative and nitrosative stress [95], prevention of pro-inflammatory cytokines [96], and serotonin levels [97] increasing. Celecoxib was largely investigated to evaluate its efficacy as an adjunctive treatment in major depression.

Several studies argue that celecoxib is effective in the management of depressive symptoms [98,99,100,101]. According to Eyre et al. [102], celecoxib is clinically inadequate. Differences in results can be attributed to the cohort heterogeneity (such as age range, gender, use in conjunction with antidepressants, the severity of depressive symptoms) [103].

In a pre-clinical animal model of depression, acetylsalicylic acid [ASA; aspirin, a selective COX-1 inhibitor] in combination with fluoxetine, a selective serotoninergic reuptake inhibitor (SSRI), seems to exert an accelerating effect. The effect of ASA (160 mg/day) augmentation therapy on SSRI in a pilot open-label study, including twenty-four non-responder patients was associated with a response rate of 52.4%. Remission was achieved in 43% of the total sample and 82% of the responder sample. In the responder group, a significant improvement was observed within week 1 and remained sustained until day 28. Despite limitations due to the open nature of this study [104], it was confirmed the accelerating effect of ASA in combination with SSRIs in the treatment of major depression (Table 1).

Simultaneously, the effect of celecoxib, which inhibits the PGE_2_ and pro-inflammatory cytokines production was studied in combination withreboxetine, a norepinephrine reuptake inhibitor (NRI) marketed as an antidepressant, on forty patients suffering from an acute depressive episode. After a wash-out period, twenty patients received 4–10 mg reboxetine plus placebo, and twenty received reboxetine plus 400 mg celecoxib for 6 weeks. There were no significant differences between groups in age, sex, duration or severity of disease or psychopathology, or reboxetine dose or plasma levels. Over 6 weeks, both groups of patients showed significant improvement in scores of the Hamilton Depression Scale (HAM-D). However, the celecoxib group showed significantly greater improvement compared to the reboxetine-alone group. Additional treatment with celecoxib has significant synergistic effects on the therapeutic action of reboxetine, improving the depressive symptomatology [105].

A six-week double-blind and placebo-controlled trial carried out to assess the efficacy of the selective COX-2 inhibitor celecoxib as an adjuvant agent in the treatment of major depression, proved that the pathophysiology of depression is associated with the hyperactivity of immune-inflammatory responses and that celecoxib reduced the production of pro-inflammatory cytokines. Thus, forty adult outpatients who met Diagnostic and Statistical Manual of Mental Disorders, 4th Edition, Text Revision (DSM-IV-TR) criteria for major depression with a baseline Hamilton Rating Scale for Depression (HRSD or HAM-D) score of at least 18 were allocated in a random fashion: 20 to fluoxetine 40 mg/day plus celecoxib 400 mg/day (200 mg bid) (morning and evening) and 20 to fluoxetine 40 mg/day plus placebo. Although both protocols significantly decreased the HRSD score over the trial period, the combination of fluoxetine and celecoxib showed a significant superiority over fluoxetine alone in the treatment of major depression symptoms. Such results suggest that celecoxib may be an effective adjuvant agent in the management of major depression patients [106].

In a randomized double-blind placebo-controlled study, forty patients with MDD and HAM-D-17 items score ≥18 were randomly assigned to either celecoxib (200 mg twice daily) or placebo in addition to SSRI sertraline (200 mg/day) for 6 weeks. The celecoxib group showed a significantly greater reduction in serum pro-inflammatory IL-6 concentration as well as HAM-D scores than the placebo group. The patients in the celecoxib group had more responses (95%) and remission (35%) than the placebo group (50% and 5%, respectively). Baseline serum IL-6 levels were significantly correlated with baseline HAM-D scores, and a significant correlation was observed between the reduction of HAM-D scores and reduction of serum IL-6 levels at week 6. Then, it was demonstrated that the antidepressant activity of celecoxib might be linked to its capability of reducing IL-6 concentration [107].

As further evidence of the beneficial effects of NSAIDs on depressive symptoms, 1497 patients affected by osteoarthritis with depressive symptoms were randomized to the placebo group, ibuprofen (800 mg 3 times daily), or naproxen (500 mg twice daily) group, or celecoxib (200 mg daily) group. Depression was assessed using the Patient Health Questionnaire-9 (PHQ-9). Median PHQ-9 score was similar in all 3 groups at baseline and after 6 weeks of treatment. Multivariable regression analysis demonstrated a detectable effect in lowering PHQ-9 scores in the ibuprofen, naproxen, and celecoxib group. The analysis shows that NSAIDs usage demonstrates a trend towards the reduction of depression symptoms in patients with osteoarthritis based upon PHQ-9 scores [108].

On thirty female outpatients diagnosed with the first episode of major depression over 8 weeks of therapy, the antidepressant effect of celecoxib (200 mg/day) augmentation of sertraline in the treatment was also studied. Participants were randomly assigned into two equal groups receiving either sertraline plus celecoxib (100 mg twice daily) or sertraline plus placebo twice daily. Patients were assessed by the Hamilton Depression and Anxiety Rating Scale at baseline, week 4, and week 8 of treatment. Celecoxib group showed a greater decrease in HAM-D scores compared to the placebo group after four weeks of treatment. Response rates were also found to be significantly higher in the celecoxib group compared to the placebo group over 4 weeks. Nevertheless, the mentioned differences between the two groups were not significant at the end of week 8. Also, the remission rate was remarkably higher in the celecoxib group in comparison with the placebo at the endpoint. The results suggested that celecoxib may hasten the onset of therapeutic action of sertraline and increase response and remission rate in depressive disorders [109].

However, NSAIDs celecoxib and naproxen on depressive symptoms in older adults recruited in Alzheimer’s Disease Anti-inflammatory Prevention Trial did not show any beneficial effects. This trial was a randomized, placebo-controlled, double-masked clinical trial conducted at six USA memory clinics. Cognitively normal volunteers age 70 and older with a family history of Alzheimer-like dementia were randomly assigned to receive celecoxib 200 mg twice daily, naproxen sodium 220 mg twice daily, or placebo. The 30-item version of the Geriatric Depression Scale (GDS) was administered to all participants at enrollment and at yearly follow-up visits. Participants with a GDS score greater than 5 at baseline were classified as depressed. Of 2528 approximately one-fifth had significant depressive symptoms at baseline. The Mean GDS score, and the percentage with significant depressive symptoms, remained similar over time across all three treatment groups. Furthermore, there was no treatment effect on GDS scores over time in the subgroup of participants with significant depressive symptoms at baseline [110].

In conclusion, the beneficial effects of NSAIDs in MDD treatment are observed only if combined with the common antidepressive drugs (fluoxetine, reboxetine, sertraline). No effects are observed by administering NSAIDs alone, as in the case of celecoxib (a selective reversible COX-2 inhibitor), ibuprofen, and naproxen (two non-selective reversible COX-1 inhibitors).

### 2.2. Schizophrenia

Schizophrenia is a chronic and extremely disabling psychiatric disorder [111,112], characterized by a wide range of symptoms classified into positive, negative, and cognitive. Positive symptoms include acoustic and visual hallucinations, paranoid delusion, and agitation, whereas negative symptoms include blunted affect, lack of volition, and disorganized speech and behavior [113,114]; cognitive symptoms include limited executive functioning, poor attention, and restricted working memory [112,115]. The onset happens during adolescence or young adulthood, and rarely in childhood [116,117]. Despite progress in treatments, most patients show persistent or varying symptoms [113,118].

The causes of schizophrenia are still mostly unknown, even if a set of evidence highlights an imbalanced network in neuroprotective/neurodegenerative factors [113,119,120]. About forty years ago, Torrey and Peterson proposed the involvement of inflammatory processes in the pathophysiology of schizophrenia [121,122]. The pro-inflammatory state in patients with schizophrenia is probably due to the interaction between environmental factors (such as infections, trauma, nutrition, and stress) and genetic vulnerability [122,123]. Several studies proved the crucial role of the immune and inflammatory processes in at least a subgroup of schizophrenic patients [124,125], giving rise to the vulnerability-stress-inflammation hypothesis of schizophrenia [126]. It focuses on the contribution of physical and mental stress in triggering schizophrenia. Accordingly, stress can represent a risk factor for the most vulnerable people (e.g., genetic factors) since it increases the levels of pro-inflammatory cytokines [127]. In addition, not only the increase of the pro-inflammatory state due to increased production of pro-inflammatory factors (TNF-α, free radicals, complement factors, and kynurenic acid) but also a decrease in the neurotrophic function of microglia and other supportive CNS cells contribute to the progressive development of schizophrenia [122,128,129]. As a result, it was observed a reduced neuronal proliferation, especially reduced connectivity and loss of brain tissue [122,130]. Furthermore, an increased brain pro-inflammatory state can also interact with glutamatergic and dopaminergic neurotransmission, thus inducing or aggravating all symptoms related to schizophrenia [122,131,132].

**Figure 9:**
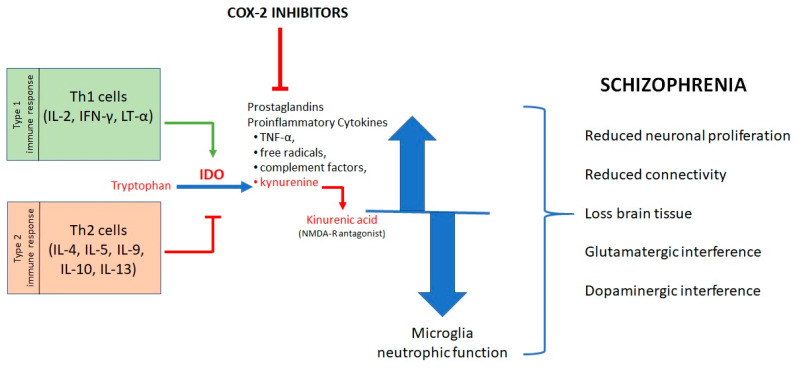
Pro-inflammatory cytokines in schizophrenia.

In this psychiatric disorder, changes in cytokine levels and an imbalance between type 1 and type 2 immune response were observed [133]. All T helper (Th) lymphocytes start out as naive Th0 cells, which, after activation, are able to “polarize,” or differentiate into either Th1 or Th2 effector cells. Mature Th1 cells secrete IL-2, IFN-γ, and LT-α, while Th2 cells secrete IL-4, IL-5, IL-9, IL-10, and IL-13. Th1 cells are principal regulators of type 1 immunity, which promote the cell-mediated immune response through lymphocytes action and are directed against intracellular pathogens. The type 2 response, instead, promotes the humoral immune response antibody-mediated [134]. Reduced production of type 1 cytokines, particularly IL-2 and IFN-γ, was found in schizophrenic patients [135,136,137]. IL-2 is involved in the regulation of dopamine release: at low concentrations promotes dopamine release, while at high concentrations inhibits it (Figure 9) [113,138].

It was observed that a reduced type 1 response occurs, mainly in the early stages of the disease, whereas an enhanced type 2 response, associated with a chronic pro-inflammatory stage, may predominate in the later stages [135]. It was also observed that dysregulation of the immune response can alter the metabolism of tryptophan. Indeed, IL-2 and IFN-γ stimulate the activity of IDO [135,139]. Conversely, a type 2 immune response inhibits the IDO catalytic activity [135]. A lower IDO activity results in higher production of kynurenic acid and in NMDA receptor antagonism [140]. It appears that reduced glutamate neurotransmission, mediated by NMDA antagonism, represents a key mechanism in the pathophysiology of schizophrenia [141,142,143,144].

The role of cytokines in schizophrenia is quite complex. In the brain, they are involved in regulating the activity of various neurotransmitters, such as serotonin, noradrenaline, dopamine, and glutamate [113]. In particular, pro-inflammatory cytokines such as IL-1β and IL-6 can affect the neuronal development of the dopaminergic and serotonergic systems [101,145,146,147,148]. Cytokines also show effects on the regulation of neuroplasticity, cell resilience, and apoptosis control [113]. TNF-α inhibits the release of the neurotrophin BDNF, thus compromising its protective effect [113]. Furthermore, a correlation between cytokine levels and negative symptoms, cognitive deficits, and psychomotor retardation was reported [101,149,150,151].

The use of anti-inflammatory agents represents a potential therapeutic approach to the treatment of schizophrenia, by virtue of the involvement of inflammation in the pathogenesis of this disease. COX-2 inhibitors show beneficial additional effects to traditional antipsychotic therapy, especially in the early stages of the disease [103,152]. These effects are due to their ability to reduce PGE_2_, type 2 cytokines, production of kynurenic acid, and to strengthen glutamate transmission [103,153].

In a prospective, double-blind evaluation, fifty patients with an acute exacerbation of schizophrenia were randomly assigned to either the antipsychotic risperidone plus celecoxib or risperidone plus placebo. After a wash-out period, twenty-five patients received 2–6 mg/day of risperidone plus placebo and twenty-five received risperidone plus 400 mg/day of celecoxib for 5 weeks. Additional treatment with celecoxib had significant positive effects on the therapeutic action of Risperidone about total schizophrenia psychopathology. Moreover, the fact that treatment with an immunomodulatory drug showed beneficial effects on schizophrenia symptoms indicates that immune dysfunction in schizophrenia is not just an epiphenomenon but is related to the pathological mechanism of the disorder [154].

The effects of celecoxib augmentation of atypical antipsychotic medications for continuously symptomatic outpatients with schizophrenia were also studied. Thirty-eight symptomatic outpatients meeting DSM-IV criteria for schizophrenia and on a stable dose of Olanzapine or Risperidone medication for at least three months were randomized to receive 8 weeks of double-blind placebo or celecoxib (400 mg/day) augmentation. In this case, celecoxib augmentation of continuously ill outpatients with schizophrenia did not improve clinical symptoms or measures of disability. This study outcome could be due to multiple factors, such as a smaller sample size than other studies and differences in the patients’ psychotic status. The participants in this study, in fact, were in more stabilized and non-acute psychotic states, as was the case with the participants in Muller’s study (Table 2).

Celecoxib efficacy, as an adjuvant agent, was also assessed in the treatment of chronic schizophrenia in an eight-week, double-blind, and placebo-controlled trial. Eligible participants in this study were sixty patients affected by chronic schizophrenia. Patients were allocated in a random fashion, 30 to risperidone 6 mg/day plus celecoxib 400 mg/day (200 mg bid) (morning and evening), and 30 to risperidone 6 mg/day plus placebo. Although both protocols significantly decreased the score of the positive, negative, and general psychopathological symptoms over the trial period, the combination of risperidone and celecoxib showed a significant superiority over risperidone alone in the treatment of positive symptoms, general psychopathology symptoms as well as Positive and Negative Syndrome Scale (PANSS) total scores. In addition, this study suggests that celecoxib may be an effective adjuvant agent in the management of patients with chronic schizophrenia (Table 2) [155].

Muller et al. started a double-blind, placebo-controlled, randomized trial by using celecoxib augmentation to amisulpride treatment in patients with the first manifestation of schizophrenia. Forty-nine patients diagnosed with schizophrenia were randomly assigned. They were treated either with amisulpride (200–1000 mg) plus celecoxib (400 mg) or amisulpride (200–1000 mg) plus a placebo. A significantly superior therapeutic effect was observed in the celecoxib group compared to placebo in the treatment of early-stage schizophrenia [156]. Zheng et al. confirmed the usefulness of celecoxib in assisting in treating the symptoms of neuropsychiatric disorders [157].

Aspirin efficacy as adjuvant treatment in schizophrenia spectrum disorders was also investigated. Seventy antipsychotic-treated inpatients and outpatients from ten psychiatric hospitals in the Netherlands with a DSM-IV-diagnosed schizophrenia spectrum disorder were included. Patients were randomized to adjuvant treatment with aspirin 1000 mg/day or placebo. During a 3-month follow-up, psychopathology was assessed with the PANSS. Aspirin given as adjuvant therapy to regular antipsychotic treatment reduces the symptoms of schizophrenia spectrum disorders. The reduction is more pronounced in those with the more altered immune function [156,157,158].

The use of NSAIDs (celecoxib, aspirin) in the treatment of schizophrenia reduces the symptoms only when administered in combination with the anti-schizophrenic drugs (i.e., risperidone, olanzapine, amisulpride). However, aspirin significantly reduced the symptoms of seventy patients in a clinical trial when administered alone.

### 2.3. Bipolar Disorder (BD)

Bipolar disorder (BD) is a chronic, complex, and debilitating psychiatric illness, resulting in alterations in mood, motility, energy levels, appetite, and sleep-alert rhythm [159,160,161,162]. It is characterized by the presence of deep and prolonged periods of depression alternating with periods of excessively high or irritable mood called mania [159]. Sometimes the transition from one phase to another is fast and immediate. Other times, it is interspersed with a period of normal mood (euthymic phase). Bipolar disorder is classified into two categories based on the level of manic symptoms: bipolar type I (BD-I) and bipolar type II (BD-II). Patients with BD-I experience at least one episode of mania, whereas patients with BD-II experience only hypomania (shorter manic episodes). Diagnosis usually occurs in late adolescence or early adulthood, although occasionally symptoms may appear as early as childhood [159]. Only about 50% of patients respond properly to psychopharmacological therapies in use, while a significant group does not respond appropriately to available alternatives [163,164,165].

Moreover, for bipolar disorder, as well as for other psychiatric illnesses, the existence of a relationship between this disease and inflammatory processes has been hypothesized. Several studies, indeed, show a dysregulation of the immune responses that manifests itself with abnormal levels of circulating pro- and anti-inflammatory cytokines in patients with BD. Serum levels of pro-inflammatory cytokines IL-4, TNF-α, soluble IL-2 receptor, IL-1b, IL-6, soluble TNF-α type 1 receptor, and C-reactive proteins are high in these subjects compared to healthy controls [162,166,167,168]. Particularly, an increase in serum levels of IL-6 and TNF was observed in the manic, euthymic, and depressive phases, while increased levels of IL-2, IL-4, and IL-8 can be observed in manic states [166,169,170]. Although there is variability in cytokine levels during depressive, manic, and euthymic periods, collected data indicate the persistence of peripheral cytokine anomalies. This finding suggests the association of BD with chronic low-grade inflammation [162,166,167,170,171,172,173,174].

The use of NSAIDs was also studied in the BD treatment (Table 3), since it was hypothesized that they have the ability to downregulate the activity of the cerebral arachidonic acid cascade by interfering with the function of Phospholipase A2 (PLA2) and/or COX [28,175,176,177,178,179,180,181,182,183].

In a clinical trial, twenty-eight BD patients who were experiencing a depressive or mixed episode, and under a stable dose of a mood stabilizer or atypical antipsychotic medication were randomized to receive 6 weeks of double-blind placebo or celecoxib (400 mg/day) treatment. From this study, celecoxib showed to produce a rapid onset of antidepressant effect in BD patients, experiencing depressive or mixed episodes [28,162,165].

Electroconvulsive therapy (ECT) is a treatment option for patients with bipolar disorder (BD). Kargar et al. studied the effect of adjunctive celecoxib on the serum cytokines of patients with BD who were undergoing ECT. This study was a randomized, double-blind, placebo-controlled trial in forty-eight patients who were diagnosed with BD and ordered to undergo six or more ECT sessions. Patients were randomly assigned to receive either placebo or celecoxib (200 mg twice daily) starting a day before the first ECT and continuing throughout the end of the sixth ECT. This study found that the level of TNF-α was significantly lower in patients receiving celecoxib compared with those on placebo at the last session of ECT. However, no significant differences in IL-1β, IL-6, and high-sensitivity C-reactive protein between the two groups were found [166].

Arabzadeh et al. started another study to demonstrate that celecoxib, via its anti-inflammatory properties, could have a therapeutic role in mood disorder. Thus, forty-six inpatients with the diagnosis of acute bipolar mania without psychotic features participated in a parallel, randomized, double-blind, placebo-controlled trial, and underwent six weeks of treatment with either celecoxib (400 mg daily) or placebo as an adjunctive treatment to sodium valproate. Patients were evaluated using the Young Mania Rating Scale (YMRS) and HRSD. The primary outcome measure with respect to efficacy was the mean decrease in the YMRS score from baseline to the study endpoint, which was compared between the two groups. A significant difference was observed in the change in YMRS scores on day 42 compared to baseline in the two groups. Celecoxib revealed to be an effective adjuvant therapy in the treatment of manic episodes (without psychotic features) of bipolar mood disorder [101,166].

Table 3 data clearly shown that the use of celecoxib powers up anti-BD drugs, improving patients’ symptomatology.

### 2.4. Autism Spectrum Disorder (ASD)

Autism spectrum disorder (ASD) is a neurodevelopmental disorder that affects communication and behavior [101,184]. It is defined as a “developmental disorder” since symptoms usually appear in the first two years of life. However, it can be diagnosed at any age [185]. According to the DSM-5, people with ASD present difficulty communicating and interacting with other people, limited interests, and repetitive behaviors [184]. Autism is known as a “spectrum” disorder because people experience a wide variety, in the type and severity, of symptoms [185]. To date, the pathogenesis of ASD still remains unknown. It is believed that alteration of brain development leading to impairment of social and communicative maturation, which manifests itself with restricted interests and repetitive behaviors [101,186]. This alteration seems to derive from processes of synaptic pruning and neuroinflammation [101,187,188,189,190].

Prenatal infections, as observed for schizophrenia, may be associated with the development of ASD [101,191,192,193]. Furthermore, both schizophrenia and ASD exhibit immune-related genetic abnormalities [101,191,194]. Abnormal activity of the glutamatergic system could play an important role in the neurotoxicity of both disorders. Abnormalities along the kynurenine pathway (Figure 6) may also be linked with 16p11.2 mutations in ASD, resulting in glutamatergic activity [101,195].

Recently, a strong inflammatory state was associated with ASD [196]. This inflammatory condition is often linked to immune system dysfunction [197]. For example, disruptions in T cells and monocytes [101,187,198], changes in immunoglobulin concentration [101,199], and autoantibody production [101,200]. Furthermore, polymorphisms identified in the macrophage migration inhibitory factor, observed in ASD-related abnormalities, also appear to activate the COX-2 system in microglia [101,201,202].

Over-activity of the immune system plays a vital role in ASD patients [203]. Several meta-analyses, indeed, demonstrated significant evidence of elevated levels of TNF-α, IFN-γ, IL-1, IL-6, IL-8, IL-12, CCL2, CCL5 e CCL11 in plasma and cerebrospinal fluid of patients affected by ASD [101,188,189,204,205,206,207]. In addition, post-mortem studies shown increased microglial density in the visual cortex, cerebellum, anterior cingulate gyrus, and dorsolateral prefrontal cortex.

One study showed that the use of repurposed anti-inflammatory agents such as pioglitazone alleviated some symptoms in ASD, such as irritability, lethargy, stereotype, and hyperactivity [208]. The class of thiazolidinediones (to which pioglitazone belongs) has been shown to inhibit COX-2 in microglia and LPS-stimulated neurons [209,210].

Asadabadi et al. [211] conducted a 10-week randomized, double-blind, placebo-controlled study on forty ASD outpatient children. These patients were randomly treated with celecoxib plus risperidone or placebo plus risperidone and assessed at baseline and after 2, 4, 6, and 10 weeks of starting medication by using the Aberrant Behavior Checklist-Community Rating Scale. In summary, the combination of Risperidone and celecoxib was superior to Risperidone alone in treating irritability, social withdrawal, and stereotypy of children with autism.

## 3. Conclusions

Numerous evidence confirms the important role that COX inhibitors play in the treatment of depression, schizophrenia, and bipolar disorder. To date, celecoxib, a selective COX-2 inhibitor, remains the most studied drug, even if some clinical trials were accomplished by using aspirin, ibuprofen, and naproxen that show a different grade of selectivity towards the two COX isoenzymes. COX inhibitors have been found to have positive effects in the treatment of psychiatric diseases when administered in combination with first-choice specific drugs. Celecoxib has been shown to hasten the onset of the effects of common therapies, on the other hand, monotherapy with COX inhibitors did not produce significant results, as it happens in some other diseases treatment.

Further studies are needed to definitively evaluate the risk/benefit ratio of these drugs and to verify whether other COX inhibitors, in addition to those already investigated, can be used in the treatment of these disorders that lower the quality of life of people who are affected.

Almost all the papers published during the last ten years were collected for this review, but as can be seen from Table 4, there are several ongoing clinical trials aimed at studying and demonstrating the efficacy of COX inhibitors as an adjuvant treatment to the usual antipsychotic therapies. It is desirable, in light of the amount of the experimentations in progress, that when all the data such studies will be available, the current state of knowledge will be consolidated and new treatments would be available for psychiatric patients.

## Figures and Tables

**Table 1:** Clinical trials investigating NSAIDs in Major Depression.

Trial	Patients	Therapy	Effects
Mendlewicz et al.	24	Fluoxetine + **aspirin**	Aspirin accelerates fluoxetine effect
Muller et al.	40	Reboxetine + **celecoxib**	Depressive symptom improvements
Akhondzadeh et al.	40	Fluoxetine + **celecoxib**	Depressive symptom improvements
Abbasi et al.	40	Sertraline + **celecoxib**	Reduction of IL-6 levels
Iyengar et al.	1497	**Ibuprofen/naproxen/celecoxib** *	Favor of NSAIDs
Majd et al.	30	Sertraline + **celecoxib**	Acceleration of sertraline therapeutic action onset/increase response and remission rate in depressive disorders
Fields et al.	2528	**Celecoxib/naproxen** *	Unfavorable results

* In this clinical trial, no antidepressive therapy was administered to the patients.

**Table 2:** Investigation of celecoxib or aspirin augmentation to anti-schizophrenic drugs.

Trial	Patients	Therapy	Effects
Muller et al.	50	Risperidone + **celecoxib**	Beneficial effects on schizophrenia symptoms
Rapaport et al.	38	Olanzapine/risperidone + **celecoxib**	No clinical symptoms improvement
Akhondzadeh et al.	60	Risperidone + **celecoxib**	Positive symptoms improvement
Muller et al.	49	Amisulpride + **celecoxib**	Superior therapeutic effect
Zheng et al.	40	Risperidone + **celecoxib**	Superior therapeutic effect
Laan et al.	70	**Aspirin**/placebo	Schizophrenia symptoms reduction

**Table 3:** Clinical trials investigating NSAIDs in Bipolar Disorder.

Trial	Patients	Therapy	Effects
Nery et al.	28	Antipsychotics + **celecoxib**	Rapid-onset antidepressant effect
Kargar et al.	48	ECT + **celecoxib**	Reduction of TNF-α levels
Arabzadeh et al.	46	Sodium valproate + **celecoxib**	Positive adjuvant effect

**Table 4:** Published and ongoing clinical trials in psychiatric disorders.

Psychiatric Disorders	Title (NCT)	Treatments/Participants	Phase	Starting Date/Recruitment Status
Depression	Biomarkers of Neuroinflammation and Anti-Inflammatory Treatments in Major Depressive Disorder (NCT02362529)	Minocycline + **celecoxib**/115	I	2015/Completed
	Late-Life Stress and Inflammation (S&I) (NCT02389465)	Escitalopram + **celecoxib**/150	IV	2014/Suspended (Study halted due to COVID-19 but it potentially will be resumed)
	Salicylic Augmentation in Depression (SAD) (NCT03152409)	**Aspirin**/74	II	2018/Recruiting
	Aspirin in Reducing Events in the Elderly (ASPREE) (NCT01038583)	**Enteric-coated aspirin**/19,114	Not Reported	2010/Active, not recruiting
Schizophrenia	A Double-blind and Randomized Trial of Celecoxib Added to Risperidone in Treatment-naive First-episode Schizophrenia(NCT00686140)	**Celecoxib**/200	NA	2006/Completed ^a^
	Efficacy and Safety of celecoxib as Add-on Therapy to Risperidone Versus Risperidone Alone in Patients With Schizophrenia (NCT00639483)	**Celecoxib**/270	II	2003/Completed ^a^
	Anti-inflammatories and Adolescent Schizophrenia (NCT04020588)	**Celecoxib** + minocycline/90	IV	2019/Recruiting
	Aspirin in Young Psychotic Patients (NCT02685748)	**Aspirin** + pantoprazole/60	III	2017/Completed ^a^
	Randomized Controlled Trial of Aspirin vs. Placebo in the Treatment of Pre-psychosis (NCT02047539)	**Aspirin**/40	Early I	2015/Recruiting
Bipolar disorder	Adjunctive Use of celecoxib in the Treatment of Bipolar Postpartum Depression (NCT02726659)	**Celecoxib**/56	III	Recruiting
	Minocycline and celecoxib as Adjunctive Treatments of Bipolar Depression (NCT02703363)	**Celecoxib** + minocycline/265	III	Completed ^a^
	Bipolar Depression and Inflammation (NCT01479829)	Escitalopram + **celecoxib**/88	IV	Completed ^a^
	Minocycline and Aspirin in the Treatment of Bipolar Depression (Minocycline) (NCT01429272)	Minocycline + **aspirin**/99	III	Completed ^a^
	*N*-Acetyl Cysteine and Aspirin as an Adjunctive Treatment for Bipolar Disorder (SMRI-Bipolar) (NCT01797575)	**Aspirin** + *N*-acetyl-cysteine/38	II	Completed ^a^

^a^ Study results are not yet posted or analyzed on ClinicalTrials.gov.

## References

[1] American Psychiatric Association. https://www.psychiatry.org/patients-families/what-is-mental-illness.

[2] Our World in Data. https://ourworldindata.org/mental-health.

[3] Kessler R.C., Amminger G.P., Aguilar-Gaxiola S., Alonso J., Lee S., Üstün T.B. (2007). Age of onset of mental disorders: A review of recent literature. Curr. Opin. Psychiatry.

[4] Haroon E., Raison C.L., Miller A.H. (2012). Psychoneuroimmunology meets neuropsychopharmacology: Translational implications of the impact of inflammation on behavior. Neuropsychopharmacology.

[5] Miller A.H., Raison L. (2015). Are anti-inflammatory therapies viable treatments for psychiatric disorders? Where the rubber meets the road. JAMA Psychiatry.

[6] Leonard B.E. (2018). Inflammation and depression: A causal or coincidental link to the pathophysiology?. Acta Neuropsychiatr..

[7] Liu Y.-Z., Wang Y.-X., Jiang C.-L. (2017). Inflammation: The common pathway of stress-related disease. Front. Hum. Neurosci..

[8] Bauer M.E., Teixeira A.L. (2018). Inflammation in psychiatric disorders: What comes first?. Ann. N. Y. Acad. Sci..

[9] Tao R., Li H. (2015). High serumuric acid level in adolescent depressive patients. J. Affect. Disord..

[10] Bartoli F., Crocamo C., Mazza M.G., Clerici M., Carrà G. (2016). Uric acid levels in subjects with bipolar disorder: A comparative meta-analysis. J. Psychiatr. Res..

[11] Oral E., Ozcan H., Kirkan T.S., Askin S., Gulec M., Aydin N. (2013). Luteal serum BDNF and HSP70 levels in women with premenstrual dysphoric disorder. Eur. Arch. Psychiatry Clin. Neurosci..

[12] Fleshner M., Frank M., Maier S.F. (2017). Danger signals and inflammasomes: Stress-evoked sterile inflammation in mood disorders. Neuropsychopharmacology.

[13] Tang D., Kang R., Coyne C.B., Zeh H.J., Lotze M.T. (2012). PAMPs and DAMPs: Signal 0s that spur autophagy and immunity. Immunol. Rev..

[14] Perrone M.G., Vitale P., Ferorelli S., Boccarelli A., Coluccia M., Pannunzio A., Campanella F., Di Mauro G., Bonaccorso C., Fortuna C.G. (2017). Effect of mofezolac-galactose distance in conjugates targeting cyclooxygenase (COX)-1 and CNS GLUT-1 carrier. Eur. J Med. Chem..

[15] Calvello R., Panaro M.A., Carbone M.L., Cianciulli A., Perrone M.G., Vitale P., Malerba P., Scilimati A. (2012). Novel selective COX-1 inhibitors suppress neuroinflammatory mediators in LPS-stimulated N13 microglial cells. Pharmacol. Res..

[16] Calvello R., Lofrumento D.D., Perrone M.G., Cianciulli A., Salvatore R., Vitale P., De Nuccio F., Giannotti L., Nicolardi G., Panaro M.A. (2017). Highly selective COX-1 inhibitors P6 and mofezolac counteract inflammatory state both in vitro and in vivo models of neuroinflammation. Front. Neurol..

[17] Tjalkens R.B., Popichak K.A., Kirkley K.A. (2017). Inflammatory activation of microglia and astrocytes in manganese neurotoxicity. Adv. Neurobiol..

[18] DiSabato D., Quan N., Godbout J.P. (2016). Neuroinflammation: The devil is in the details. J. Neurochem..

[19] Miller B.J., Buckley P., Seabolt W., Mellor A., Kirkpatrick B. (2011). Meta-analysis of cytokine alterations in schizophrenia: Clinical status and antipsychotic effects. Biol. Psychiatry..

[20] Meyer U., Feldon J., Yee B.K. (2009). A review of the fetal brain cytokine imbalance hypothesis of schizophrenia. Schizophr. Bull..

[21] Muller N., Myint A.M., Schwarz M.J. (2012). Inflammation in schizophrenia. Adv. Protein Chem. Struct. Biol..

[22] Miller B.J., Culpepper N., Rapaport M.H., Buckley P. (2013). Prenatal inflammation and neurodevelopment in schizophrenia: A review of human studies. Prog. Neuro Psychopharmacol. Biol. Psychiatry.

[23] Potvin S., Stip E., Sepehry A.A., Gendron A., Bah R., Kouassi E. (2008). Inflammatory cytokine alterations in schizophrenia: A systematic quantitative review. Biol. Psychiatry.

[24] Drexhage R.C., Hoogenboezem T.A., Cohen D., Versnel M.A., Nolen W.A., van Beveren N.J.M., Drexhage H.A. (2011). An activated set point of T-cell and monocyte inflammatory networks in recent-onset schizophrenia patients involves both pro- and anti-inflammatory forces. Int. J. Neuropsychopharmacol..

[25] Berk M., Kapczinski F., Andreazza A.C., Dean O.M., Giorlando F., Maes M., Yücel M., Gama C.S., Dodd S., Dean B. (2011). Pathways underlying neuroprogression in bipolar disorder: Focus on inflammation, oxidative stress and neurotrophic factors. Neurosci. Biobehav. Rev..

[26] Drexhage R.C., Knijff E.M., Padmos R.C., van der Heul-Nieuwenhuijzen L., Beumer W., Versnel M.A., Drexhage H.A. (2010). The mononuclear phagocyte system and its cytokine inflammatory networks in schizophrenia and bipolar disorder. Expert Rev. Neurother..

[27] Goldstein B.I., Kemp D.E., Soczynska J.K., McIntyre R.S. (2009). Inflammation and the phenomenology, pathophysiology, comorbidity, and treatment of bipolar disorder: A systematic review of the literature. J. Clin. Psychiatry.

[28] Fond G., Hamdani N., Kapczinski F., Boukouaci W., Drancourt N., Dargel A., Oliveira J., Guen E.L., Marlinge E., Tamouza R. (2014). Effectiveness and tolerance of anti-inflammatory drugs’ add-on therapy in major mental disorders: A systematic qualitative review. Acta Psychiatr. Scand..

[29] Taams L.S. (2019). Neuroimmune interactions: How the nervous and immune systems influence each other. Clin. Exp. Immunol..

[30] Norris G.T., Kipnis J. (2019). Immune cells and CNS physiology: Microglia and beyond. J. Exp. Med..

[31] Hopkins S.J. (2007). Central nervous system recognition of peripheral inflammation: A neural, hormonal collaboration. Acta Biomed.

[32] Smith W.L., Urade Y., Jakobsson P. (2011). Enzymes of the cyclooxygenase pathways of prostanoid biosynthesis. Chem. Rev..

[33] Ricciotti E., Garret A.F. (2011). Prostaglandins and inflammation. Arterioscler. Thromb. Vasc. Biol..

[34] Kam P.C.A., See A.U.L. (2000). Cyclo-oxygenase isoenzymes: Physiological and pharmacological role. Anaesthesia.

[35] Kaufmann W.E., Worley P.F., Pegg J., Bremer M., Isakson P.C. (1996). COX-2, a synaptically induced enzyme, is expressed by excitatory neurons at postsynaptic sites in rat cerebral cortex. Proc. Natl. Acad. Sci. USA.

[36] Kaufmann W.E., Andreasson K.I., Isakson P.C., Worley P.F. (1997). Cyclooxygenases and the central nervous system. Prostaglandins.

[37] Maes M. (2012). Targeting cyclooxygenase-2 in depression is not a viable therapeutic approach and may even aggravate the pathophysiology underpinning depression. Metab. Brain Dis..

[38] Stefanovic B., Bosetti F., Silva A.C. (2006). Modulatory role of cyclooxygenase-2 in cerebrovascular coupling. Neuroimage.

[39] Hewett S.J., Bell S.C., Hewett J.A. (2006). Contributions of cyclooxygenase-2 to neuroplasticity and neuropathology of the central nervous system. Pharmacol. Ther..

[40] Liu Z., Cheng X., Zhong S., Zhang X., Liu C., Liu F., Zhao C. (2020). Peripheral and central nervous system immune response crosstalk in amyotrophic lateral sclerosis. Front. Neurosci..

[41] Burian M., Geisslinger G. (2005). COX-dependent mechanisms involved in the antinociceptive actionof NSAIDs at central and peripheral sites. Pharmacol. Ther..

[42] Rostevanov I.S., Boyko M., Ferorelli S., Scilimati A., Perrone M.G., Kaplanski J., Zlotnik A., Azab A.N. (2020). Inhibition of cyclooxygenase-1 does not reduce mortality in post-ischemic stroke rats. Neurosci. Lett..

[43] Rao P.P.N., Knaus E.E. (2008). Evolution of nonsteroidal anti-inflammatory drugs (NSAIDs): Cyclooxygenase (COX) inhibition and beyond. J. Pharm. Pharm. Sci..

[44] Perrone M.G., Scilimati A., Simone L., Vitale P. (2010). Selective COX-1 inhibition: A therapeutic target to be reconsidered. Curr. Med. Chem..

[45] Ajmone-Cat M.A., Bernardo A., Greco A., Minghetti L. (2010). Non-steroidal anti-inflammatory drugs and brain inflammation: Effects on microglial functions. Pharmaceuticals.

[46] Novakova I., Subileau E.-A., Toegel S., Gruber D., Lachmann B., Urban E., Chesne C., Noe C.R., Neuhaus W. (2014). Transport rankings of non-steroidal antiinflammatory drugs across blood-brain barrier in vitro models. PLoS ONE.

[47] Berthold-Losleben M., Heitmann S., Himmerich H. (2009). Anti-inflammatory drugs in psychiatry. Inflamm. Allergy Drug Targets.

[48] Riolo S.A., Nguyen T.A., Greden J.F., King C.A. (2005). Prevalence of depression by race/ethnicity: Findings from the National Health and Nutrition Examination Survey III. Am. J. Public Health.

[49] Kessler R.C., McGonagle K.A., Zhao S., Nelson C.B., Hughes M., Eshleman S., Wittchen H.U., Kendler K.S. (1994). Lifetime and 12-month prevalence of DSM-III-R psychiatric disorders in the United States. Results from the National Comorbidity Survey. Arch. Gen. Psychiatry.

[50] Trivedi M.H., Rush A.J., Wisniewski S.R., Nierenberg A.A., Warden D., Ritz L., Norquist G., Howland R.H., Lebowitz B., McGrath P.J. (2006). Evaluation of outcomes with citalopram for depression using measurement-based care in STAR*D: Implications for clinical practice. Am. J. Psychiatry.

[51] Zhang J., Yao W., Hashimoto K. (2016). Brain-derived neurotrophic factor (BDNF)-TrkB signaling in inflammation-related depression and potential therapeutic targets. Curr. Neuropharmacol..

[52] Smith R.S. (1991). The macrophage theory of depression. Med. Hypotheses.

[53] Feltes P.K., Doorduin J., Klein H.C., Juárez-Orozco L.E., Dierckx R.A., Moriguchi-Jeckel C.M., de Vries E.F.J. (2017). Anti-inflammatory treatment for major depressive disorder: Implications for patients with an elevated immune profile and non-responders to standard antidepressant therapy. J. Psychopharmacol..

[54] Dantzer R., O’Connor J.C., Lawson M.A., Kelley K.W. (2011). Inflammation-associated depression: From serotonin to kynurenine. Psychoneuroendocrinology.

[55] Köhler O., Krogh J., Mors O., Benros M.E. (2016). Inflammation in depression and the potential for anti-inflammatory treatment. Curr. Neuropharmacol..

[56] Howren M.B., Lamkin D.M., Suls J. (2009). Associations of depression with C-reactive protein, IL-1, and IL-6: A meta-analysis. Psychosom. Med..

[57] Benros M.E., Waltoft B.L., Nordentoft M., Ostergaard S.D., Eaton W.W., Krogh J., Mortensen P.B. (2013). Autoimmune diseases and severe infections as risk factors for mood disorders: A nationwide study. JAMA Psychiatry.

[58] Dantzer R., Capuron L., Irwin M.R., Miller A.H., Ollat H., Perry V.H., Rousey S., Yirmiya R. (2008). Identification and treatment of symptoms associated with inflammation in medically ill patients. Psychoneuroendocrinology.

[59] Dantzer R., O’Connor J.C., Freund G.G., Johnson R.W., Kelley K.W. (2008). From inflammation to sickness and depression: When the immune system subjugates the brain. Nat. Rev. Neurosci..

[60] Pucak M.L., Kaplin A.I. (2005). Unkind cytokines: Current evidence for the potential role of cytokines in immune-mediated depression. Int. Rev. Psychiatry.

[61] Maes M., Bosmans E., Suy E., Vandervorst C., DeJonckheere C., Raus J. (1991). Depression-related disturbances in mitogen-induced lymphocyte responses and interleukin-1 beta and soluble interleukin-2 receptor production. Acta Psychiatr. Scand..

[62] Dowlati Y., Herrmann N., Swardfager W., Liu H., Sham L., Reim E.K., Lanctôt K.L. (2010). A meta-analysis of cytokines in major depression. Biol. Psychiatry.

[63] Dahl J., Ormstad H., Aass H.C., Malt U.F., Bendz L.T., Sandvik L., Brundin L., Andreassen O.A. (2014). The plasma levels of various cytokines are increased during ongoing depression and are reduced to normal levels after recovery. Psychoneuroendocrinology.

[64] Liu Y., Ho R.C., Mak A. (2012). Interleukin (IL)-6, tumour necrosis factor alpha (TNF-α) and soluble interleukin-2 receptors (sIL-2R) are elevated in patients with major depressive disorder: A metaanalysis and meta-regression. J. Affect. Disord..

[65] Mikova O., Yakimova R., Bosmans E., Kenis G., Maes M. (2001). Increased serum tumor necrosis factor alpha concentrations in major depression and multiple sclerosis. Eur. Neuropsychopharmacol..

[66] Berk M., Wadee A.A., Kuschke R.H., O’Neill-Kerr A. (1997). Acute phase proteins in major depression. J. Psychosom. Res..

[67] Wium-Andersen M.K., Ørsted D.D., Nielsen S.F., Nordestgaard B.G. (2013). Elevated C-reactive protein levels, psychological distress, and depression in 73, 131 individuals. JAMA Psychiatry.

[68] Maes M., Scharpé S., Van Grootel L., Uyttenbroeck W., Cooreman W., Cosyns P., Suy E. (1992). Higher alpha 1-antitrypsin, haptoglobin, ceruloplasmin and lower retinol binding protein plasma levels during depression: Further evidence for the existence of an inflammatory response during that illness. J Affect. Disord..

[69] Kim Y.K., Na K.S., Shin K.H., Jung H.Y., Choi S.H., Kim J.B. (2007). Cytokine imbalance in the pathophysiology of major depressive disorder. Prog. Neuro Psychopharmacol. Biol. Psychiatry.

[70] Krishnadas R., Cavanagh J. (2012). Depression: An inflammatory illness?. J. Neurol. Neurosurg. Psychiatry.

[71] El-Sisi A.E.-S., Sokkar S.S., El-Sayad M.E.-S., Ramadan E.S., Osman E.Y. (2016). Celecoxib and omega-3 fatty acids alone and in combination with risperidone affect the behavior and brain biochemistry in amphetamine-induced model of schizophrenia. Biomed. Pharmacother..

[72] Liu H., Luiten P.G.M., Eisel U.L.M., Dejongste M.J.L., Schoemaker R.G. (2013). Depression after myocardial infarction: TNF-α-induced alterations of the blood-brain barrier and its putative therapeutic implications. Neurosci. Biobehav. Rev..

[73] Baune B.T. (2017). Are non-steroidal anti-inflammatory drugs clinically suitable for the treatment of symptoms in depression-associated inflammation?. Curr. Top. Behav. Neurosci..

[74] Ruhe H.G., Mason N.S., Schene A.H. (2007). Mood is indirectly related to serotonin, norepinephrine and dopamine levels in humans: A meta-analysis of monoamine depletion studies. Mol. Psychiatry.

[75] Satyanarayana U., Narasinga Rao B.S. (1980). Dietary tryptophan level and the enzymes of tryptophan NAD pathway. Br. J. Nutr..

[76] Carlin J.M., Borden E.C., Sondel P.M., Byrne G.I. (1989). Interferon-induced indoleamine 2,3-dioxygenase activity in human mononuclear phagocytes. J. Leukoc. Biol..

[77] Taylor M.W., Feng G. (1991). Relationship between interferon-γ, indoleamine 2,3-dioxygenase, and tryptophan catabolism. FASEB J..

[78] Gałecki P., Talarowska M. (2018). Inflammatory theory of depression. Psychiatr. Pol..

[79] Anderson G. (2016). Editorial: The kynurenine and melatonergic pathways in psychiatric and CNS disorders. Curr. Pharm. Des..

[80] Wirleitner B., Neurauter G., Schröcksnadel K., Frick B., Fuchs D. (2003). Interferon-gamma-induced conversion of tryptophan: Immunologic and neuropsychiatric aspects. Curr. Med. Chem..

[81] Myint A.M., Kim Y.K. (2003). Cytokine-serotonin interaction through IDO: A neurodegeneration hypothesis of depression. Med. Hypotheses.

[82] Guillemin G.J., Wang L., Brew B.J. (2005). Quinolinic acid selectively induces apoptosis of human astocytes: Potential role in AIDS dementia complex. J. Neuroinflammation.

[83] Guillemin G.J., Smythe G., Takikawa O., Brew B.J. (2005). Expression of indoleamine 2,3-dioxygenase and production of quinolinic acid by human microglia, astrocytes, and neurons. Glia.

[84] Spalletta G., Bossù P., Ciaramella A., Bria P., Caltagirone C., Robinson R.G. (2006). The etiology of poststroke depression: A review of the literature and a new hypothesis involving inflammatory cytokines. Mol. Psychiatry.

[85] Lehrmann E., Guidetti P., Löve A., Williamson J., Bertram E.H., Schwarcz R. (2008). Glial activation precedes seizures and hippocampal neurodegeneration in measles virus-infected mice. Epilepsia.

[86] Muller N., Schwarz M.J. (2007). The immune-mediated alteration of serotonin and glutamate: Towards an integrated view of depression. Mol. Psychiatry.

[87] Dunn A.J., Swiergiel A.H., De Beaurepaire R. (2005). Cytokines as mediators of depression: What can we learn from animal studies?. Neurosci. Biobehav. Rev..

[88] Zhu C.-B., Blakely R.D., Hewlett W.A. (2006). The proinflammatory cytokines interleukin-1beta and tumor necrosis factor-alpha activate serotonin transporters. Neuropsychopharmacology.

[89] Malynn S., Campos-Torres A., Moynagh P., Haase J. (2013). The pro-inflammatory cytokine TNF-α regulates the activity and expression of the serotonin transporter (SERT) in astrocytes. Neurochem. Res..

[90] Cai W., Khaoustov V.I., Xie Q., Pan T., Le W., Yoffe B. (2005). Interferon-α-induced modulation of glucocorticoid and serotonin receptors as a mechanism of depression. J. Hepatol..

[91] Pariante C.M. (2003). Depression, stress and the adrenal axis. J. Neuroendocrinol..

[92] Stellwagen D., Beattie E.C., Seo J.Y., Malenka R.C. (2005). Differential regulation of AMPA receptor and GABA receptor trafficking by tumor necrosis factor-α. J. Neurosci..

[93] Raison C.L., Miller A.H. (2003). When not enough is too much: The role of insufficient glucocorticoid signaling in the pathophysiology of stress-related disorders. Am. J. Psychiatry.

[94] Schuld A., Schmid D.A., Haack M., Holsboer F., Friess E., Pollmächer T. (2003). Hypothalamo-pituitary-adrenal function in patients with depressive disorders is correlated with baseline cytokine levels, but not with cytokine responses to hydrocortisone. J. Psychiatr. Res..

[95] Anderson G., Berk M., Dean O., Moylan S., Maes M. (2014). Role of immune-inflammatory and oxidative and nitrosative stress pathways in the etiology of depression: Therapeutic implications. CNS Drugs.

[96] Casolini P., Catalani A., Zuena A.R., Angelucci L. (2002). Inhibition of COX-2 reduces the age-dependent increase of hippocampal inflammatory markers, corticosterone secretion, and behavioral impairments in the rat. J. Neurosci. Res..

[97] Sandrini M., Vitale G., Pini L.A. (2002). Effect of rofecoxib on nociception and the serotonin system in the rat brain. Inflamm. Res..

[98] Köhler C.A., Freitas T.H., Maes M., de Andrade N.Q., Liu C.S., Fernandes B.S., Stubbs B., Solmi M., Veronese N., Herrmann N. (2017). Peripheral cytokine and chemokine alterations in depression: A meta-analysis of 82 studies. Acta Psychiatr. Scand..

[99] Köhler O., Benros M.E., Nordentoft M., Farkouh M.E., Iyengar R.L., Mors O., Krogh J. (2014). Effect of anti-inflammatory treatment on depression, depressive symptoms, and adverse effects a systematic review and meta-analysis of randomized clinical trials. JAMA Psychiatry.

[100] Na K.S., Lee K.J., Lee J.S., Cho Y.S., Jung H.Y. (2014). Efficacy of adjunctive celecoxib treatment for patients with major depressive disorder: A meta-analysis. Prog. Neuro Psychopharmacol. Biol. Psychiatry.

[101] Sethi R., Gómez-Coronado N., Walker A.J., Robertson O.D., Agustini B., Berk M., Dodd S. (2019). Neurobiology and therapeutic potential of cyclooxygenase-2 (COX-2) inhibitors for inflammation in neuropsychiatric disorders. Front. Psychiatry.

[102] Eyre H.A., Air T., Pradhan A., Johnston J., Lavretsky H., Stuart M.J., Baune B.T. (2016). A meta-analysis of chemokines in major depression. Prog. Neuro. Psychopharmacol. Biol. Psychiatry.

[103] Yui K., Imataka G., Nakamura H., Ohara N., Naito Y. (2015). Eicosanoids derived from arachidonic acid and their family prostaglandins and cyclooxygenase in psychiatric disorders. Curr. Neuropharmacol..

[104] Mendlewicz J., Kriwin P., Oswald P., Souery D., Alboni S., Brunello N. (2006). Shortened onset of action of antidepressants in major depression using acetylsalicylic acid augmentation: A pilot open-label study. Int. Clin. Psychopharmacol..

[105] Müller N., Schwarz M.J., Dehning S., Douhe A., Cerovecki A., Goldstein-Müller B., Spellmann I., Hetzel G., Maino K., Kleindienst N. (2006). The cyclooxygenase-2 inhibitor celecoxib has therapeutic effects in major depression: Results of a double-blind, randomized, placebo controlled, add-on pilot study to reboxetine. Mol. Psychiatry.

[106] Akhondzadeh S., Jafari S., Raisi F., Nasehi A.A., Ghoreishi A., Salehi B., Mohebbi-Rasa S., Raznahan M., Kamalipour A. (2009). Clinical trial of adjunctive celecoxib treatment in patients with major depression: A double blind and placebo controlled trial. Depress. Anxiety.

[107] Abbasi S.H., Hosseini F., Modabbernia A., Ashrafi M., Akhondzadeh S. (2012). Effect of celecoxib add-on treatment on symptoms and serum IL-6 concentrations in patients with major depressive disorder: Randomized double-blind placebo-controlled study. J. Affect. Disord..

[108] Iyengar R.L., Gandhi S., Aneja A., Thorpe K., Razzouk L., Greenberg J., Mosovich S., Farkouh M.E. (2013). NSAIDs are associated with lower depression scores in patients with osteoarthritis. Am. J. Med..

[109] Majd M., Hashemian F., Hosseinib S.M., Shariatpanahi M.V., Sharifid A. (2015). A randomized, double-blind, placebo-controlled trial of celecoxib augmentation of sertraline in treatment of drug-naive depressed women: A pilot study. Iran. J. Pharm. Res..

[110] Fields C., Drye L., Vaidya V., Lyketsos C. (2012). Celecoxib or naproxen treatment does not benefit depressive symptoms in persons age 70 and older: Findings from a randomized controlled trial. Am. J. Geriatr. Psychiatry.

[111] World Health Organization Schizophrenia [Fact Sheet]. www.who.int/en/news-room/fact-sheets/detail/schizophrenia9April2018.

[112] Schmidt L., Phelps E., Friedel J., Shokraneh F. (2019). Acetylsalicylic acid (Aspirin) for schizophrenia. Cochrane Database Syst. Rev..

[113] Mansur R.B., Zugman A., Asevedo E.D.M., Da Cunha G.R., Bressan R.A., Brietzke E. (2012). Cytokines in schizophrenia: Possible role of anti-inflammatory medications in clinical and preclinical stages. Psychiatry Clin. Neurosci..

[114] Tandon R., Gaebel W., Barch D.M., Bustillo J., Gur R.E., Heckers S., Malaspina D., Owen M.J., Schultz S., Tsuang M. (2013). Definition and description of schizophrenia in the DSM-5. Schizophr. Res..

[115] Simpson E.H., Kellendonk C., Kandel E. (2010). A possible role for the striatum in the pathogenesis of the cognitive symptoms of schizophrenia. Neuron.

[116] Shi J., Levinson D.F., Duan J., Sanders A.R., Zheng Y., Péer I., Dudbridge F., Holmans P.A., Whittemore A.S., Mowry B.J. (2009). Common variants on chromosome 6p22.1 are associated with schizophrenia. Nature.

[117] Keller W.R., Kum L.M., Wehring H.J., Koola M.M., Buchanan R.W., Kelly D.L. (2013). A review of anti-inflammatory agents for symptoms of schizophrenia. J. Psychopharmacol..

[118] WHO (2001). Burden of mental and behavioral disorders. Mental Health: New Understanding, New Hope.

[119] Lieberman J.A., Buckley P.F., Perkins D.O. (2007). Neuroprotection: A new strategy in the treatment of schizophrenia. CNS Spectr..

[120] Ono T., Hashimoto E., Ukai W., Ishii T., Saito T. (2010). The role of neural stem cells for in vitro models of schizophrenia: Neuroprotection via Akt/ERK signal regulation. Schizophr. Res..

[121] Torrey E.F., Peterson M.R. (1973). Slow and latent viruses in schizophrenia. Lancet.

[122] Sommer I.E., Van Westrhenen R., Begemann M.J.H., De Witte L.D., Leucht S., Kahn R.S. (2014). Efficacy of anti-inflammatory agents to improve symptoms in patients with schizophrenia: An update. Schizophr. Bull..

[123] Fineberg A.M., Ellman L.M. (2013). Inflammatory cytokines and neurological and neurocognitive alterations in the course of schizophrenia. Biol. Psychiatry.

[124] Müller N. (2019). COX-2 inhibitors, aspirin, and other potential anti-inflammatory treatments for psychiatric disorders. Front. Psychiatry.

[125] Sekar A., Bialas A.R., De Rivera H., Davis A., Hammond T.R., Kamitaki N., Tooley K., Presumey J., Baum M., Van Doren V. (2016). Schizophrenia risk from complex variation of complement component 4. Nature.

[126] Zubin J., Spring B. (1977). Vulnerability: A new view of schizophrenia. J. Abnorm. Psychol..

[127] Müller N. (2016). What role does inflammation play in schizophrenia?. Expert Rev. Neurother..

[128] Monji A., Kato T., Kanba S. (2009). Cytokines and schizophrenia: Microglia hypothesis of schizophrenia. Psychiatry Clin. Neurosci..

[129] Drexhage R.C., Weigelt K., van Beveren N., Cohen D., Versnel M.A., Nolen W.A., Drexhage H.A. (2011). Immune and neuroimmune alterations in mood disorders and schizophrenia. Int. Rev. Neurobiol..

[130] Chew L.J., Fusar-Poli P., Schmitz T. (2013). Oligodendroglial alterations and the role of microglia in white matter injury: Relevance to schizophrenia. Int. J. Dev. Neurosci..

[131] Müller N., Schwarz S.M. (2006). Schizophrenia as an inflammation-mediated dysbalance of glutamatergic neurotransmission. Neurotox. Res..

[132] Muller N., Dursun S.M. (2011). Schizophrenia genes, epigenetics and psychoneuroimmunology therapeutics: All make sense now?. J. Psychopharmacol..

[133] Chiang S.S.W., Riedel M., Schwarz M., Mueller N. (2013). Is T-helper type 2 shift schizophrenia-specific? Primary results from a comparison of related psychiatric disorders and healthy controls. Psychiatry Clin. Neurosci..

[134] Spellberg B., Edwards J.E. (2001). Type 1/type 2 immunity in infectious diseases. Clin. Infect. Dis..

[135] Müller N., Schwarz M.J. (2010). Immune system and schizophrenia. Curr. Immunol. Rev..

[136] Wilke I., Arolt V., Rothermundt M., Weitzsch C., Hornberg M., Kirchner H. (1996). Investigations of cytokine production in whole blood cultures of paranoid and residual schizophrenic patients. Eur. Arch. Psychiatry Clin. Neurosci..

[137] Müller N., Riedel M., Ackenheil M., Schwarz M.J. (2000). Cellular and humoral immune system in schizophrenia: A conceptual re-evaluation. World J. Biol. Psychiatry.

[138] Petitto J.M., McCarthy D.B., Rinker C.M., Huang Z., Getty T. (1997). Modulation of behavioral and neurochemical measures of forebrain dopamine function in mice by species-specific interleukin-2. J. Neuroimmunol..

[139] Grohmann U., Fallarino F., Puccetti P. (2003). Tolerance, DCs and tryptophan: Much ado about IDO. Trends Immunol..

[140] Attari A., Mojdeh A., Soltani F.A.S.K., Najarzadegan M.R. (2017). Aspirin inclusion in antipsychotic treatment on severity of symptoms in schizophrenia: A randimized clinical trial. Iran. J. Psychiatry Behav. Sci..

[141] Müller N., Schwarz M.J. (2007). The immunological basis of glutamatergic disturbance in schizophrenia: Towards an integrated view. J. Neural Transm..

[142] Genius J., Geiger J., Dölzer A.L., Benninghoff J., Giegling I., Hartmann A.M., Möller H.J., Rujescu D. (2013). Glutamatergic dysbalance and oxidative stress in in vivo and in vitro models of psychosis based on chronic NMDA receptor antagonism. PLoS ONE.

[143] Howes O., McCutcheon R., Stone J. (2015). Glutamate and dopamine in schizophrenia: An update for the 21st century. J. Psychopharmacol..

[144] Müller N., Weidinger E., Leitner B., Schwarz M.J. (2015). The role of inflammation in schizophrenia. Front. Neurosci..

[145] Jarskog L.F., Xiao H., Wilkie M.B., Lauder J.M., Gilmore J.H. (1997). Cytokine regulation of embryonic rat dopamine and serotonin neuronal survival in vitro. Int. J. Dev. Neurosci..

[146] Kabiersch A., Del Rey H.F.A., Besedovsky H.O. (1998). Administration of interleukin-1 at birth affects dopaminergic neurons in adult mice. Ann. N. Y. Acad. Sci..

[147] Ling Z.D., Potter E.D., Lipton J.W., Carvey P.M. (1998). Differentiation of mesencephalic progenitor cells into dopaminergic neurons by cytokines. Exp. Neurol..

[148] Potter E.D., Ling Z.D., Carvey P.M. (1999). Cytokine-induced conversion of mesencephalic-derived progenitor cells into dopamine neurons. Cell Tissue Res..

[149] Holden J.M., Meyers-Manor J.E., Overmier J.B., Gahtan E., Sweeney W., Miller H. (2008). Lipopolysaccharide-induced immune activation impairs attention but has little effect on short-term working memory. Behav. Brain Res..

[150] Möller H.J. (2007). Clinical evaluation of negative symptoms in schizophrenia. Eur. Psychiatry.

[151] Tandon R., Nasrallah H.A., Keshavan M.S. (2009). Schizophrenia, “just the facts” 4. Clinical features and conceptualization. Schizophr. Res..

[152] Muller N., Myint A.-M., Schwarz M.J. (2012). Immunological treatment options for schizophrenia. Curr. Pharm. Biotechnol..

[153] Schmidt L., Ceglarek U., Kortz L., Hoop M., Kirkby K., Thiery J., Himmerich H. (2013). Mechanisms of involvement of eicosanoids and their precursors in the pathophysiology and treatment of schizophrenia. Med. Chem..

[154] Müller N., Riedel M., Scheppach C., Brandstätter B., Sokullu S., Krampe K., Ulmschneider M., Engel R.R., Möller H.J., Schwarz M.J. (2002). Beneficial antipsychotic effects of celecoxib add-on therapy compared to risperidone alone in schizophrenia. Am. J. Psychiatry.

[155] Akhondzadeh S., Tabatabaee M., Amini H., Ahmadi Abhari S.A., Abbasi S.H., Behnam B. (2007). Celecoxib as adjunctive therapy in schizophrenia: A double-blind, randomized and placebo-controlled trial. Schizophr. Res..

[156] Müller N., Krause D., Dehning S., Musil R., Schennach-Wolff R., Obermeier M., Möller H.J., Klauss V., Schwarz M.J., Riedel M. (2010). Celecoxib treatment in an early stage of schizophrenia: Results of a randomized, double-blind, placebo-controlled trial of celecoxib augmentation of amisulpride treatment. Schizophr. Res..

[157] Zheng W., Cai D.-B., Yang X.H., Ungvari G.S., Ng C.H., Müller N., Ning Y.P., Xiang Y.T. (2017). Adjunctive celecoxib for schizophrenia: A meta-analysis of randomized, double-blind, placebo-controlled trials. J. Psychiatr. Res..

[158] Laan W., Grobbee D.E., Selten J.P., Heijnen C.J., Kahn R.S., Burger H. (2010). Adjuvant aspirin therapy reduces symptoms of schizophrenia spectrum disorders: Results from a randomized, double-blind, placebo-controlled trial. J. Clin. Psychiatry.

[159] National Institute of Mental Health. https://www.nimh.nih.gov/health/topics/bipolar-disorder/index.shtml.

[160] Kupfer D.J. (2005). The increasing medical burden in bipolar disorder. JAMA.

[161] Fagiolini A., Forgione R., Maccari M., Cuomo A., Morana B., Dell’Osso M.C., Pellegrini F., Rossi A. (2013). Prevalence, chronicity, burden and borders of bipolar disorder. J. Affect. Disord..

[162] Rosenblat J.D., Kakar R., Berk M., Kessing L.V., Vinberg M., Baune B.T., Mansur R.B., Brietzke E., Goldstein B.I., Mcintyre R.S. (2016). Anti-inflammatory agents in the treatment of bipolar depression: A systematic review and meta-analysis. Bipolar Disord..

[163] Gershon S., Soares J.C. (1997). Current therapeutic profile of lithium. Arch. Gen. Psychiatry.

[164] Soares J.C. (2000). Recent advances in the treatment of bipolar mania, depression, mixed states, and rapid cycling. Int. Clin. Psychopharmacol..

[165] Nery F.G., Monkul E.S., Hatch J.P., Fonseca M., Zunta-Soares G.B., Frey B.N., Bowden C.L., Soares J.C. (2008). Celecoxib as an adjunct in the treatment of depressive or mixed episodes of bipolar disorder: A double-blind, randomized, placebo-controlled study. Hum. Psychopharmacol..

[166] Arabzadeh S., Ameli N., Zeinoddini A., Rezaei F., Farokhnia M., Mohammadinejad P., Ghaleiha A., Akhondzadeh S. (2015). Celecoxib adjunctive therapy for acute bipolar mania: A randomized, double-blind, placebo-controlled trial. Bipolar Disord..

[167] Barbosa I.G., Bauer M.E., MacHado-Vieira R., Teixeira A.L. (2014). Cytokines in bipolar disorder: Paving the way for neuroprogression. Neural Plast..

[168] Munkholm K., Braüner J.V., Kessing L.V., Vinberg M. (2013). Cytokines in bipolar disorder vs. healthy control subjects: A systematic review and meta-analysis. J. Psychiatr. Res..

[169] Modabbernia A., Taslimi S., Brietzke E., Ashrafi M. (2013). Cytokine alterations in bipolar disorder: A meta-analysis of 30 studies. Biol. Psychiatry.

[170] Leza J.C., Bueno B., Bioque M., Arango C., Parellada M., Do K., O’Donnell P., Bernardo M. (2015). Inflammation in schizophrenia: A question of balance. Neurosci. Biobehav. Rev..

[171] Barbosa I.G., Machado-Vieira R., Soares J.C., Teixeira A.L. (2014). The immunology of bipolar disorder. Neuroimmunomodulation.

[172] Barbosa I.G., Rocha N.P., Bauer M.E., De Miranda A.S., Huguet R.B., Reis H.J., Zunszain P.A., Horowitz M.A., Pariante C.M., Teixeira A.L. (2013). Chemokines in bipolar disorder: Trait or state?. Eur. Arch. Psychiatry Clin. Neurosci..

[173] Munkholm K., Vinberg M., Vedel Kessing L. (2013). Cytokines in bipolar disorder: A systematic review and meta-analysis. J. Affect. Disord..

[174] Munkholm K., Weikop P., Kessing L.V., Vinberg M. (2015). Elevated levels of IL-6 and IL-18 in manic and hypomanic states in rapid cycling bipolar disorder patients. Brain Behav. Immun..

[175] Brietzke E., Kauer-Sant’Anna M., Teixeira A.L., Kapczinski F. (2009). Abnormalities in serum chemokine levels in euthymic patients with bipolar disorder. Brain Behav. Immun..

[176] Duncan R.E., Bazinet R.P. (2010). Brain arachidonic acid uptake and turnover: Implications for signaling and bipolar disorder. Curr. Opin. Clin. Nutr. Metab. Care.

[177] Rao J.S., Rapoport S.I. (2009). Mood-stabilizers target the brain arachidonic acid cascade. Curr. Mol. Pharm..

[178] Bazinet R.P. (2009). Is the brain arachidonic acid cascade a common target of drugs used to manage bipolar disorder?. Biochem. Soc. Trans..

[179] Rapoport S.I., Basselin M., Kim H.W., Rao J.S. (2009). Bipolar disorder and mechanisms of action of mood stabilizers. Brain Res. Rev..

[180] Rapoport S.I. (2008). Brain arachidonic and docosahexaenoic acid cascades are selectively altered by drugs, diet and disease. Prostaglandins Leukot. Essent. Fat. Acids.

[181] Rao J.S., Lee H.J., Rapoport S.I., Bazinet R.P. (2008). Mode of action of mood stabilizers: Is the arachidonic acid cascade a common target?. Mol. Psychiatry.

[182] Van Strater A.C.P., Bouvy P.F. (2007). Omega-3 fatty acids in the treatment of affective disorders: An overview of the literature. Tijdschr. Psychiatr..

[183] Quiroz J.A., Gould T.D., Manji H.K. (2004). Molecular effects of lithium. Mol. Interv..

[184] (2013). Diagnostic and Statistical Manual of Mental Disorders: DSM-5.

[185] National Institute of Mental Health. https://www.nimh.nih.gov/health/topics/autism-spectrum-disorders/index.shtml.

[186] Muhle R., Trentacoste S.V., Rapin I. (2004). The genetics of autism. Pediatrics.

[187] Goines P., Van De Water J. (2010). The immune system’s role in the biology of autism. Curr. Opin. Neurol..

[188] Nakagawa Y., Chiba K. (2016). Involvement of neuroinflammation during brain development in social cognitive deficits in autism spectrum disorder and schizophrenia. J. Pharmacol. Exp. Ther..

[189] Onore C., Careaga M., Ashwood P. (2012). The role of immune dysfunction in the pathophysiology of autism. Brain Behav. Immun..

[190] Petrelli F., Pucci L., Bezzi P. (2016). Astrocytes and microglia and their potential link with autism spectrum disorders. Front. Cell. Neurosci..

[191] Brown A.S. (2012). Epidemiologic studies of exposure to prenatal infection and risk of schizophrenia and autism. Dev. Neurobiol..

[192] Fang S.Y., Wang S., Huang N., Yeh H.H., Chen C.Y. (2015). Prenatal infection and autism spectrum disorders in childhood: A population-based case-control study in Taiwan. Paediatr. Perinat. Epidemiol..

[193] Jiang H.-Y., Xu L.-L., Shao L., Xia R.-M., Yu Z.-H., Ling Z.-X., Yang F., Deng M., Ruan B. (2016). Maternal infection during pregnancy and risk of autism spectrum disorders: A systematic review and meta-analysis. Brain Behav. Immun..

[194] Ziats M.N., Rennert O.M. (2011). Expression profiling of autism candidate genes during human brain development implicates central immune signaling pathways. PLoS ONE.

[195] Lim C.K., Essa M.M., de Paula Martins R., Lovejoy D.B., Bilgin A.A., Waly M.I., Al-Farsi Y.M., Al-Sharbati M., Al-Shaffae M.A., Guillemin G.J. (2016). Altered kynurenine pathway metabolism in autism: Implication for immune-induced glutamatergic activity. Autism Res..

[196] Croonenberghs J., Bosmans E., Deboutte D., Kenis G., Maes M. (2002). Activation of the inflammatory response system in autism. Neuropsychobiology.

[197] Brigida A.L., Schultz S., Cascone M., Antonucci N., Siniscalco D. (2017). Endocannabinod signal dysregulation in autism spectrum disorders: A correlation link between inflammatory state and Neuro-Immune alterations. Int. J. Mol. Sci..

[198] Ashwood P., Krakowiak P., Hertz-Picciotto I., Hansen R., Pessah I., Van de Water J. (2011). Elevated plasma cytokines in autism spectrum disorders provide evidence of immune dysfunction and are associated with impaired behavioral outcome. Brain Behav. Immun..

[199] Heuer L., Ashwood P., Schauer J., Goines P., Krakowiak P., Hertz-Picciotto I., Hansen R., Croen L.A., Pessah I.N., Van De Water J. (2008). Reduced levels of immunoglobulin in children with autism correlateswith behavioral symptoms. Autism Res..

[200] Wills S., Cabanlit M., Bennett J., Ashwood P., Amaral D., Van De Water J. (2007). Autoantibodies in autism spectrum disorders (ASD). Ann. N. Y. Acad. Sci..

[201] Grigorenko E.L., Han S.S., Yrigollen C.M., Leng L., Mizue Y., Anderson G.M., Mulder E.J., De Bildt A., Minderaa R.B., Volkmar F.R. (2008). Macrophage migration inhibitory factor and autism spectrum disorders. Pediatrics.

[202] Wang F.Z., Wu H.B., Xu S.Q., Guo X.R., Yang J., Shen X.F. (2011). Macrophage migration inhibitory factor activates cyclooxygenase 2-prostaglandin E2 in cultured spinal microglia. Neurosci. Res..

[203] Friedrich M.J. (2014). Research on psychiatric disorders targets inflammation. JAMA.

[204] Vargas D.L., Nascimbene C., Krishnan C., Zimmerman A.W., Pardo C.A. (2005). Neuroglial activation and neuroinflammation in the brain of patients with autism. Ann. Neurol..

[205] Careaga M., Schwartzer J., Ashwood P. (2015). Inflammatory profiles in the BTBR mouse: HOW relevant are they to autism spectrum disorders?. Brain Behav. Immun..

[206] El-Ansary A., Al-Ayadhi L. (2012). Neuroinflammation in autism spectrum disorders. J. Neuroinflammation.

[207] Masi A., Quintana D.S., Glozier N., Lloyd A.R., Hickie I.B., Guastella A.J. (2015). Cytokine aberrations in autism spectrum disorder: A systematic review and meta-analysis. Mol. Psychiatry.

[208] Boris M., Kaiser C.C., Goldblatt A., Elice M.W., Edelson S.M., Adams J.B., Feinstein D.L. (2007). Effect of pioglitazone treatment on behavioral symptoms in autistic children. J. Neuroinflammation.

[209] Kim E.J., Kwon K.J., Park J.Y., Lee S.H., Moon C.H., Baik E.J. (2002). Effects of peroxisome proliferator-activated receptor agonists on LPS-induced neuronal death in mixed cortical neurons: Associated with iNOS and COX-2. Brain Res..

[210] Sepanjnia K., Modabbernia A., Ashrafi M., Modabbernia M.J., Akhondzadeh S. (2012). Pioglitazone adjunctive therapy for moderate-to-severe major depressive disorder: Randomized double-blind placebo-controlled trial. Neuropsychopharmacology.

[211] Asadabadi M., Mohammadi M.R., Ghanizadeh A., Modabbernia A., Ashrafi M., Hassanzadeh E., Forghani S., Akhondzadeh S. (2013). Celecoxib as adjunctive treatment to risperidone in children with autistic disorder: A randomized, double-blind, placebo-controlled trial. Psychopharmacology.

